# Endothelin-Dependent Vasoconstriction in Human Uterine Artery: Application to Preeclampsia

**DOI:** 10.1371/journal.pone.0016540

**Published:** 2011-01-26

**Authors:** Clotilde Dechanet, Aurélie Fort, Elisabet Barbero-Camps, Hervé Dechaud, Sylvain Richard, Anne Virsolvy

**Affiliations:** 1 INSERM U637, Université Montpellier 1 and 2, Montpellier, France; 2 Service de gynécologie-obstétrique, CHU A. de Villeneuve, Montpellier, France; Institute of Zoology, Chinese Academy of Sciences, China

## Abstract

**Background:**

Reduced uteroplacental perfusion, the initiating event in preeclampsia, is associated with enhanced endothelin-1 (ET-1) production which feeds the vasoconstriction of uterine artery. Whether the treatments of preeclampsia were effective on ET-1 induced contraction and could reverse placental ischemia is the question addressed in this study. We investigated the effect of antihypertensive drugs used in preeclampsia and of ET receptor antagonists on the contractile response to ET-1 on human uterine arteries.

**Methodology/Principal Findings:**

Experiments were performed, *ex vivo*, on human uterine artery samples obtained after hysterectomy. We studied variations in isometric tension of arterial rings in response to the vasoconstrictor ET-1 and evaluated the effects of various vasodilators and ET-receptor antagonists on this response. Among antihypertensive drugs, only dihydropyridines were effective in blocking and reversing the ET-1 contractile response. Their efficiency, independent of the concentration of ET-1, was only partial. Hydralazine, alpha-methyldopa and labetalol had no effect on ET-1 induced contraction which is mediated by both ET_A_ and ET_B_ receptors in uterine artery. ET receptors antagonists, BQ-123 and BQ-788, slightly reduced the amplitude of the response to ET-1. Combination of both antagonists was more efficient, but it was not possible to reverse the maximal ET-1-induced contraction with antagonists used alone or in combination.

**Conclusion:**

Pharmacological drugs currently used in the context of preeclampsia, do not reverse ET-1 induced contraction. Only dihydropyridines, which partially relax uterine artery previously contracted with ET-1, might offer interesting perspectives to improve placental perfusion.

## Introduction

Preeclampsia (PE) is a pregnancy-specific vasoconstrictive disorder, characterized by the development of hypertension and proteinuria after 20 weeks of gestation in previously normotensive, non-proteinuric women [1]. Hypertension, which develops during pregnancy, remits spontaneously after delivery.

PE is associated with abnormal placentation consecutive to alterations in the vascular remodeling of uteroplacental arteries mainly due to abnormal invasion of trophoblasts and unconverted narrow spiral arteries [2]. These processes are the cause of underperfusion and persistent hypoxia in the placenta [3], [4] leading to an imbalanced production of vasoactive factors [5]. Thus, the placenta is thought to release various mediators into the maternal circulation, which causes local endothelial dysfunction and vasoconstriction of uterine arteries [6], and contributes to systemic blood pressure elevation [7]. Among these substances, endothelin-1 (ET-1), a potent vasoactive peptide produced by endothelial cells, appears to be increased [8]–[10] thereby promoting vasospasms and impairing uteroplacental blood flow [11], [12]. Elevation of plasma concentrations of ET-1, is involved in the vasoconstriction of uterine artery associated with the syndrome [10], [13], [14] and contributes to systemic hypertension [15]. Blood pressure and ET-1 concentration rapidly return to normal levels within 48 h after delivery of the placenta.

Current treatments for PE are aimed at normalizing blood pressure rather than targeting the pathology itself, but none is really satisfactory. In most cases, hypertension is reduced only transiently, allowing a caesarean delivery to be set up before term. The main treatment recommendations include the use of antihypertensive drugs such as the calcium (Ca^2+^) channel blockers of the dihydropyridine class (DHP) (nifedipine or nicardipine), vasodilators (e.g. labetalol or hydralazine), antihypertensive (methyldopa) and magnesium sulfate [16].

If ET-1 plays a central role in the vasoconstriction of uterine arteries, and consequently in the reduced uteroplacental perfusion and the progression of PE, targeting the contractile response to ET-1 of uteroplacental arteries could be an effective therapeutic strategy offering interesting perspectives for the management of the pathology.

In the present work, we investigated the effect of various drugs used in the treatment of PE, to inhibit the vasoconstrictive response of the human uterine artery to ET-1. In particular, we considered a new option in the management of PE based on the use of selective ET-1 receptor antagonists (ERAs), and we evaluated the contribution of DHP-sensitive Ca^2+^ channels to the vasoconstrictive response to ET-1.

## Materials and Methods

### Tissue collection

The study was approved by the French ministry of research and higher education (DC-2008-488) and local ethics committee (CPP Sud Mediterranee-IV) with informed consent in writing from each patient. Specimens of uterine artery were obtained from thirty normotensive non-pregnant women (mean age 44 years, range 35–53 years), undergoing hysterectomy for benign gynecological disorders. None of the selected patients were on hormone therapy or any other medication and had history of cardiovascular disease. Immediately after the removal of the uterus, the main branch of one uterine artery was excised from the parametrium, connective tissues and adjacent myometrium, placed in physiological saline buffer (PSS; composition in mM: 140 NaCl, 5 KCl, 1 MgCl_2_, 0.5 KH_2_PO_4_, 0.5 Na_2_HPO_4_, 2.5 CaCl_2_, 10 HEPES, 10 glucose, pH 7.4) and transported to the laboratory within 30 minutes.

### Drugs

The following compounds were used and all purchased from Sigma-Aldrich: human ET-1, sarafotoxin-S6c (selective ET_B_ receptor agonist), BQ-123 (selective ET_A_ receptor antagonist), BQ-788 (selective ET_B_ receptor antagonist), nifedipine and nicardipine (DHP class of calcium channel blocker), hydralazine, labetalol (alpha/beta blocker) and alpha-methyldopa (á_2_-adrenergic agonist). Compounds were dissolved either in water or in DMSO and stored as 10 mM or 1 mM stock solutions. Final molar concentrations in the organ bath are indicated when necessary in the text. The final concentration of DMSO did not exceed 0.1% and had no effect on vasoconstriction.

### Vascular reactivity

Artery specimens were dissected under a binocular microscope, isolated from paracervical connective tissues and sixteen segments of 2–3 mm length were cut. The outer diameter of the vessels was 2 to 3 mm. The endothelium was removed by rubbing and artery segments were mounted onto thin stainless steel holders and placed in conventional 5 ml organ bath chambers filled with PSS, maintained at 37°C and continuously bubbled with O_2_. Changes in isometric tension were recorded using an IT1-25 force transducer and an IOX computerized system (EMKA Technologies, Paris, France). Experiments were performed as previously described [17]. Arterial segments were subjected to a 60 min equilibration period at the predetermined optimal point of its active length-tension curve established at 2 g by measuring the contraction to 30 mM potassium chloride (KCl) at different levels of stretch. The contractile function of each segment and a maximal contraction was determined with 10 ìM phenylephrine (Phe). Preparations were discarded if they failed to contract to Phe or exhibited spontaneous contractile activity. The removal of endothelium was confirmed by subsequent application of 1 µM acetylcholine (Ach); in the absence of endothelium, contractility is not modified. After a wash (W) and 20–30 minutes period of stabilization, arterial ring responses to endothelin (ET-1) were studied. For experiments performed in the presence of antagonists and vasodilators, preparations were subjected to a 20 min incubation period with the agent before the addition of ET-1. Dose-response curves were generated by cumulative increases in the concentration of ET-1 (range 0.1 nM to 0.5 µM) and normalized to the contraction induced by Phe (10 µM) in the same preparation. Relaxing effects of antagonists and antihypertensive drugs were evaluated in arteries contracted with ET-1 (0.1 µM). When the contraction was established, the agent was added and the tension was then compared to the response to ET-1. For each compound, the concentrations used corresponded to doses maximally active. Hydralazine, alpha-methyldopa and labetalol were used at 100 µM; BQ-123, BQ-788, sarafotoxin-S6c, nifedipine and nicardipine at 1 µM. At a pharmacological level, these concentrations corresponded to that maximally active for each drug. Specific protocols are detailed when necessary in the legends.

### Data analysis

All data are expressed as means ± standard error of the mean (S.E.M.), for *n* specimen of uterine artery. For each specimen, protocols were performed in triplicate on different arterial rings. Data were statistically analyzed using one-way ANOVA followed by a Dunnett post-test. P-values lower than 0.05 were considered significant. Analyses were performed using GraphPad Prism® (Graph-Pad Software Inc., San Diego, USA) and EC_50_ values were determined using the same software from data fitted with a non-linear function.

## Results

### Contractile response to ET-1

**Figure 1:**
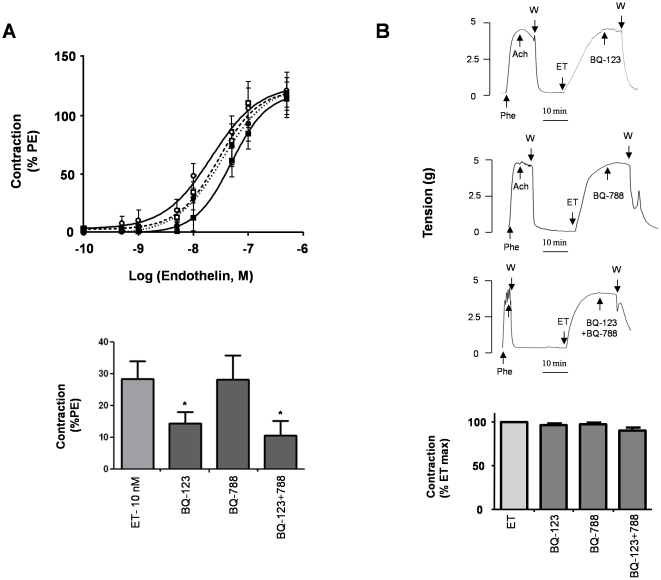
Contractile response of the human uterine artery to ET-1 and effect of ET receptor antagonists. (**A**) Upper panel: Dose-response curves obtained by cumulative increases in the concentration of ET-1 (0.1 nM–0.1 µM) on basal tone (CTL,❍), after pre-incubation for 10 minutes with either an ET_A_ receptor antagonist (BQ-123, 1 µM,•) or an ET_B_ receptor antagonist (BQ-788, 1 µM,☐), or a mixture of both antagonists (BQ-123+788, 1 µM,▪). Lower panel: Average contraction of uterine artery induced by 10 nM ET-1 with or without a 10 minutes pre-incubation period with either BQ-123, BQ-788 or BQ123+788 each at 1 µM concentration. Data are expressed as a percentage of the contraction induced by Phe (10 µM). (**B**) Effects of ET receptor antagonists when arteries were contracted with ET-1 (0.1 µM). When maximal contraction was established, ET receptor antagonists were added at the same concentrations used in panel A as illustrated on typical recordings of variations of isometric tension (upper panels). Bar graph represents the average tension after the addition of the drugs, expressed as a percentage of the contraction induced by ET-1 (lower panel). In all experiments, values indicate the means ± S.E.M. of 10 different arteries with experiments performed in triplicate.

ET-1 caused a potent and long lasting contraction of the human uterine artery. The contraction developed slowly, was sustained after reaching its maximal amplitude (Fig. 1), was reversed only partially by the removal of the agonist and could not be induced again (not shown). The ET-1 induced vasoconstriction was dose-dependent, with a threshold at approximately 1 nM (Fig. 1A). The maximal amplitude of 6.7±1 g, obtained at 0.5 µM ET-1, was similar to the maximal effect of phenylephrine (10 µM), which induced a tension of 5.8±0.7 g (n = 10).

Treatment with selective ERAs (BQ-123 for ET_A_ and BQ-788 for ET_B_) did not change the baseline tone of uterine arteries but did modify the effect of ET-1 (Fig. 1A). BQ-123 (1 µM) and BQ-788 (1 µM) competitively antagonized the contraction induced by ET-1, whereas the amplitude of the contraction elicited by a maximally active dose of the vasoconstrictor was not affected. The dose-response curves of ET-1 were slightly shifted to the right by both antagonists individually and by a combination of the two. The EC_50_ value of ET-1 (24.5±5.6 nM, n = 10) was increased after pre-treatment with BQ-123 (33.9±1.8 nM, p = 0.03, n = 10) but not with BQ-788 (28.8±2.2 nM, p = 0.48, n = 10). The application of BQ-123 and BQ-788 in combination inhibited the response to ET-1 more efficiently (EC_50_ = 48.9±1.3 nM, p = 0.0001, n = 10) (Fig. 1A, upper panel). The response to 10 nM ET-1 was reduced by 50% by BQ-123, and by 62% by the two antagonists applied together (Fig. 1A, lower panel). For a saturating concentration of ET-1 (0.5 µM), the inhibitory effects of antagonists represent only 5% of control values. The two ERAs alone or in combination did not modify the amplitude of the contraction induced by the maximal dose of ET-1 (0.5 µM) (Fig. 1A). BQ-123 and BQ-788, added either alone or in combination, did not relax arteries pre-contracted with 0.1 µM ET-1 (Fig. 1B).

### Effect of the ET_B_ receptor agonist sarafotoxin

**Figure 2:**
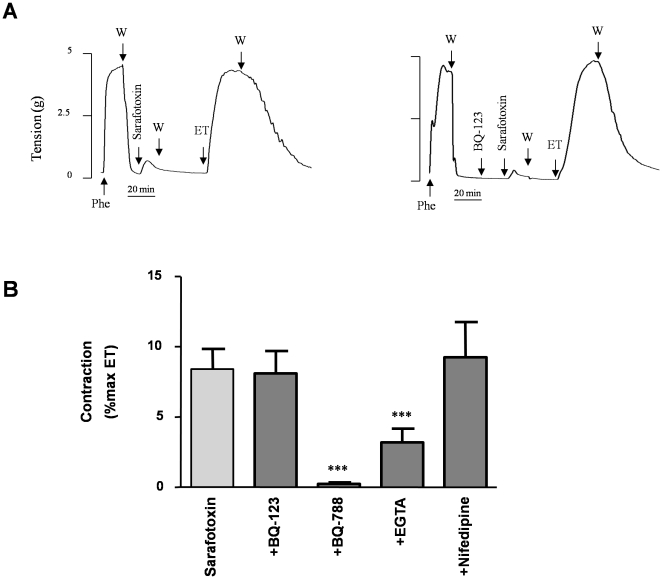
Effect of sarafotoxin S6c on uterine artery contraction. The effect of sarafotoxin S6c (1 µM) was analyzed with or without a 10-minute pre-incubation period with either 1 µM BQ-123, BQ-788, nifedipine or 10 mM EGTA, and compared to the contraction induced by ET-1 alone (0.1 µM). (**A**) Representative recordings of isometric tension changes illustrating the effect of sarafotoxin S6c in presence (right panel) or in absence (left panel) of BQ-123 (other drugs not shown). (**B**) Average contraction obtained with sarafotoxin S6c under basal conditions (CTL) or in the presence of BQ-123, BQ-788, EGTA or nifedipine. Data, expressed as a percentage of the contraction induced by ET-1 (0.1 µM) in the same experiment, indicate the means ± S.E.M. of 10 different arteries, with experiments performed in triplicate. *** p<0.0001.

The selective ET_B_ agonist Sarafotoxin-S6c elicited the vasoconstriction of human uterine arteries (Fig. 2). The maximal contractile response was weak, reaching only 8.4±1.4% (n = 10) of the maximal response to ET-1. This agonistic effect was fully inhibited by the ET_B_ receptor antagonist BQ-788 but was unaffected by the ET_A_ receptor antagonist BQ-123 (Fig. 2B). The contraction induced by sarafotoxin-S6c was unaltered by the DHP nifedipine (1 µM) but was decreased by the extracellular application of EGTA (10 mM), a Ca^2+^-chelating agent (Fig. 2B).

### Antagonism of ET-1 induced contraction

**Figure 3:**
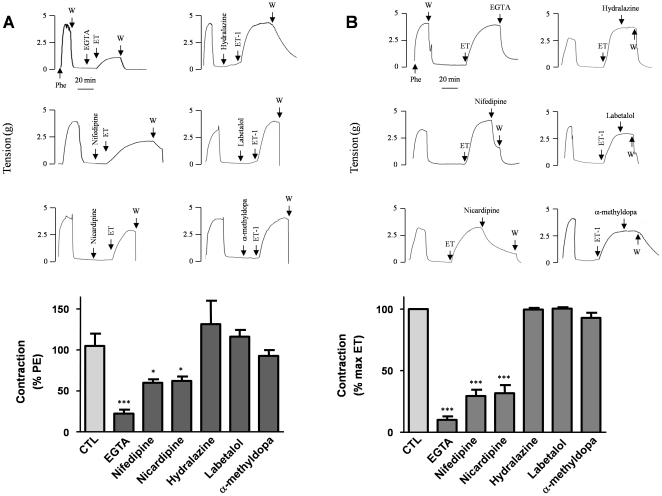
Antagonism of the contraction induced by ET-1. Upper panels represent typical recordings of isometric tension for each condition. Lower panels are bar graph representations of the respective data. (**A**) A 10-minute pre-incubation was carried out before contraction was elicited by ET-1 (0.1 µM), with either EGTA (10 mM), nifedipine or nicardipine (1 µM), or hydralazine, labetalol or alpha-methyldopa (100 µM). Data are expressed as a percentage of the contraction induced by Phe and represent the maximal tension reached with ET-1 in the presence of the drugs. (**B**) The artery was contracted with ET-1 (0.1 µM); derivatives were added when the steady state was established. Data represent the residual tension after the addition of drugs and are expressed as a percentage of the maximal contraction induced by ET-1. Values indicate the means ± S.E.M. of 10 different arteries, with experiments performed in triplicate. *p<0.01, ***p<0.0001.

Hydralazine, labetalol and alpha-methyldopa (100 µM) did not affect the contractile response to ET-1 (Fig. 3A) and did not reverse the contraction induced by ET-1 (Fig. 3B).

**Figure 4:**
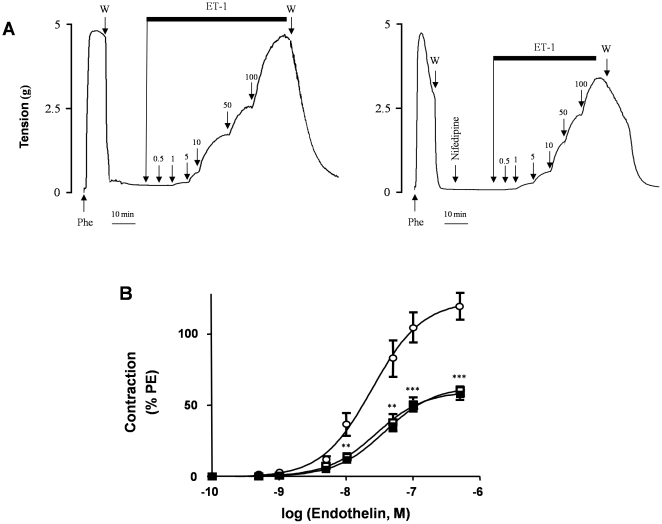
Effect of dihydropyridines on the contractile response of the uterine artery to ET-1. ET-1 concentration was cumulatively increased (0.1 nM–0.1 µM) with or without a 10-minute pre-incubation period with a DHP. (**A**) Representative recordings of isometric responses. (**B**) Dose response curves obtained for ET-1 in the absence (❍) or in the presence of nifedipine (1 µM,☐) or nicardipine (1 µM,▪). Data, expressed as a percentage of the contraction induced by Phe in the same experiment, indicate the means ± S.E.M. of 10 different arteries with experiments performed in triplicate. **p<0.001, ***p<0.0001.

Pretreatment with the Ca^2+^-chelating agent EGTA (10 mM) reduced the amplitude of ET-1-induced uterine artery contraction (Fig. 3A). The contractile response to 0.1 µM ET-1 was 22.3±4.9% of control levels without EGTA. Furthermore, 10 mM EGTA reversed the contraction elicited by 0.1 µM ET-1 by 90±2.9% (p<0.0001, n = 10) (Fig. 3B). After pretreatment with the Ca^2+^ channel antagonists nifedipine (1 µM) or nicardipine (1 µM), the effects of the maximal dose of ET-1 (0.5 µM) were reduced respectively by 39.9±4% (p<0.005, n = 10) and by 37.6±5.2% (p<0.005, n = 10) (Fig. 3A). The same concentrations of the DHPs also relaxed arteries pre-contracted with ET-1 (0.1 µM), reversing the contraction by 70.5±5.1% in the case of nifedipine and by 68.3±6.6% in the case of nicardipine (Fig. 3B). In addition, both DHPs partially inhibited the effect of ET-1 at all active concentrations (Fig. 4). The dose-response curve was slightly shifted to the right: the EC_50_ increased from 24.5±5.6 nM in controls to 38.2±2.5 nM (p = 0.003, n = 10) with nifedipine and to 27.9±4.8 nM (p = 0.021, n = 10) with nicardipine. At all ET-1 concentrations, the amplitude of the full contractile response was reduced on average by 50% with nifedipine and with nicardipine. No difference was observed in terms of efficacy between nifedipine and nicardipine.

## Discussion

ET-1 induced a strong and sustained contraction of the human uterine artery, which was mediated by both ET_A_ and ET_B_ receptors. ERAs prevented the contractile response to low ET-1 concentration, but failed either to reduce contraction when ET-1 levels were high or to relax uterine arteries pre-contracted with ET-1, even when they were combined. Classical antihypertensive treatments used in PE did not either reverse or prevent ET-1 induced contraction in human uterine artery except for DHPs, consistently with the involvement of voltage-gated Ca^2+^ channels in the development and, predominantly, in the maintenance of this response. These results provide an experimental basis to interpret the failure of PE treatments and to propose a rationale, based on the use of DHPs, for a potential approach in the management of the pathology.

Since experiments were performed in endothelium denuded uterine arteries, our results are consistent with the presence of ET-1 receptors at the VSMC level. The EC_50_ value of ET-1 effect (24.5±5.6 nM) is in line with previous findings in these and other vessels [18], [19]. The weak agonistic effect of sarafotoxin-S6C and the inhibitory effect of ET_B_R antagonists, show that ET_B_R also contributes to this regulation even if the vasopressor response to ET-1 seems to be mainly mediated by ET_A_R. Synergistic inhibition by a combination of ERAs, already described for other blood vessels [20], [21], supports the idea that ET_A_R and ET_B_R are simultaneously involved in ET-1-induced contraction of uterine artery.

Signal transduction pathways for ET-1 related to receptor subtypes distribution vary between vascular beds and species [22]. In the human uterine artery, the ET-1-induced contraction depended predominantly (>70%) on Ca^2+^ entry, as shown by experiments in Ca^2+^-free conditions with EGTA. Several types of Ca^2+^ channels could be involved in this regulation, especially the L-type Ca^2+^ channels. Nifedipine and nicardipine, the Ca^2+^ channel antagonists of choice in PE, blocked half of the ET-1 response at micromolar concentration, supporting the idea that DHP-sensitive L-type Ca^2+^ channels are involved specifically. The partial inhibition by DHPs together with the effect of EGTA suggest that ET-1 also activates DHP-insensitive Ca^2+^ influx, which might be through voltage-insensitive receptor-operated channels (ROCs) or non-selective cation channels (NSCC) [23]. Studies performed on myometrial [24] and on uteroplacental arteries [25] showed a more pronounced relaxation of ET-1 induced contraction with DHPs, but the high doses used (up to 100 µM) are not consistent with a specific and selective action on L-type Ca^2+^channels [26].

We observed that hydralazine, labetalol and alpha-methyldopa, antihypertensive drugs used in the treatment of PE, did not modify either the contraction induced by ET-1 or the response of artery to the vasoconstrictor. This suggests that, even if they reduce the systemic hypertension *in vivo*, these drugs are inefficient to antagonize the ET-1 induced vasospasm of uterine artery. Consistently, hydralazine, labetalol and alpha-methyldopa do not change uteroplacental blood flow in hypertensive pregnant women [27]–[29].

Our data also suggest that targeting ET-1 receptors is not a good option. ET_A_R and ET_B_R antagonists prevented ET-1-induced contraction only partially even with a maximally active combination of both derivatives. Additionally, when ET-1 concentration is maximal, the inhibition of its effect is barely significant, in accordance with the action of reversible competitive antagonists. Moreover, antagonists, applied individually or in combination, were unable to relax ET-1-precontracted arteries, which could be explained by the rapid internalization of ET-1/ETR complexes making the ET-1 binding site no longer accessible to the ERAs [30]. Our data on uterine artery are in line with studies performed on myometrial resistance arteries also showing a partial inhibition of ET-1 induced contraction though contraction is more pronounced in preeclamptic specimen [31]. Besides the fact that they are considered teratogenic [32] and contraindicated in pregnancy [33], ERAs are not expected to do better than Ca^2+^ antagonists.

The first aim in the treatment of PE is to control severe acute hypertension. Treatment is usually administered when mild to moderate hypertension is detected during pregnancy, in order to delay or halt the evolution toward severe hypertension, but none achieves this goal. ET-1 induced vasoconstriction of uterine artery is supposed to be one contributing factor leading to blood pressure elevation in PE. We show here that, among treatments used in PE, only DHPs reverse the contraction of the uterine artery induced by ET-1 although it does so partially. While these derivatives are commonly used [34], they do not prevent the progression of the pathology [7] and although the prolongation of pregnancy is better with DHPs, as compared to hydralazine and labetalol, the occurrence of delivery is still premature [35]. One reason could be that, by the time systemic hypertension occurs, ET-1 concentrations have reached maximal saturating levels in the placenta [36] not reflected by the slight elevation in plasma levels [37]. Chronic placental hypoxia/ischemia enhances local ET-1 expression [38], [39], these increases are likely to promote the vasospastic activity of uterine arteries and thus maintain a vicious cycle of hypoxia/ischemia and ET-1 production. In this situation, no improvement is expected in placental perfusion as no relaxation of ET-1 induced vasoconstriction occurs. Consistent with the effects of antihypertensive drugs on the reactivity of uterine artery, only acute antihypertensive treatments with DHPs are expected to lower systemic blood pressure. However, they may not sufficiently relax uterine arteries maximally constricted, and thus may be ineffective with respect to placental perfusion. Abnormal uteroplacental blood flow is revealed in uterine artery Doppler ultrasonography by an increased pulsatility index and a bilateral notching [40], [41]. Thus, as soon as Doppler investigation identifies abnormalities during the first or second trimester of pregnancy, a therapy based on the use of DHP derivatives might be set up. DHPs administrated earlier, *i.e.* when ET-1 concentrations are not maximal, are expected to stop the vicious cycle (vasoconstriction *vs.* ET-1 production).

One limitation of this study is the use of uterine arteries from non pregnant and non preeclamptic women. For obvious ethical and practical reasons, it is not possible to have access to these arteries either in PE or in pregnancy. In PE, vascular resistance is increased and it is unlikely to be the consequence of altered responses to vasoconstrictors. Contractile responses of vascular smooth muscle to vasoactive agonists and sensitivity of contraction to changes in intracellular [Ca^2+^] does not seem to be affected by the pathology [42], [43]. Even if vascular reactivity is altered, the ET-1 system is constitutively activated in the pathology as shown by the lack of effect of exogenous ET-1 in preeclamptic patients and the down regulation of receptor expression due to the high levels of ET-1 in uteroplacental circulation [44], [45]. Our data show no or only partial effect of drugs on uterine artery when the ET-1 system is activated. Conclusions raised on the basis of pharmacological mechanisms involving ET-1 signaling pathway are relevant in that context. Moreover, our observations are in line with the limited clinical efficiency of the drugs currently used in PE. Labetalol, hydralazine and alpha methyldopa, efficient for systemic hypertension, have only transient lowering effect on blood pressure and do not prevent the progression to severe hypertension. DHPs targeting the mechanisms downstream of ET-1 signaling may offer more appropriate therapeutic options in that context.

In conclusion, our data provide some mechanistic insights to explain the partial failure of PE treatment with antihypertensive drugs. We showed that hydralazine, labetalol and alpha-methyldopa do not antagonize the vasoconstriction of uterine artery induced by ET-1 and, so, they are not supposed to stop the self-sustained process of vasoconstriction and imbalanced production of vasoactive mediators. We do not expect them to slow the progression of the pathology. DHPs were the most interesting drugs providing that they are prescribed earlier, ideally before the onset of severe hypertension. Only under these conditions DHP derivatives can significantly improve placental perfusion, reverse placental hypoxia and delay the onset of PE.
